# “This is what a war does”- Trust, information ecosystems and childhood vaccination among Ukrainian parents: A qualitative study

**DOI:** 10.1371/journal.pgph.0006742

**Published:** 2026-07-02

**Authors:** Harriet Dwyer, Nadine Beckmann, Jennifer Palmer, Fedir Lapii, Dorota Kleszczewska, Agnieszka Sochon-Latuszek, Hanna Yahorava, Kateryna Gatsenko, Luisa Enria

**Affiliations:** 1 Department of Global Health and Development, London School of Hygiene and Tropical Medicine, London, United Kingdom; 2 Department of Paediatrics, Immunology of Infectious and Rare Diseases, European Medical School, First European University, Kyiv, Ukraine; 3 Department of Health Sociology, Education and Medical Communication, Institute of Mother and Child, Warsaw, Poland; 4 Institute of Mother and Child Foundation, Warsaw, Poland; 5 BeChange Poland, Warsaw, Ukraine; 6 Independent Research Assistants/Freelance Journalists; University of Greenwich, UNITED KINGDOM OF GREAT BRITAIN AND NORTHERN IRELAND

## Abstract

Armed conflict not only disrupts health systems but reconfigures trust in vaccination and interactions with information. This study explored how the war in Ukraine has reshaped vaccine attitudes among parents living in Lviv, Ukraine and those displaced to Warsaw, Poland. Using a qualitative design, we conducted semi-structured interviews with 30 Ukrainian parents and 21 key informants, including healthcare workers, journalists, humanitarian actors and policy makers. Data were collected between November 2024 and March 2025 and analysed thematically to identify key patterns in how conflict and displacement influence trust and decision-making around childhood vaccination. Findings show that war and displacement disrupted continuity of care, with vaccination often deprioritised as families navigate documentation, housing and safety. Structural and language barriers further limited access, especially for displaced families. Yet, some parents described vaccination as a means to protect their children and reclaim their agency amid uncertainty, demonstrating active engagement with healthcare despite instability. Information practices were also reconfigured: digital platforms, particularly Telegram, became key spaces for seeking, sharing and interpreting information. Trust was shaped by perceived authenticity, responsiveness and emotional connection to information sources. This study highlights that access and vaccine confidence are mutually reinforcing. At the same time, efforts to sustain trust in vaccination must therefore go beyond focusing on information (and mis/disinformation) and draw on empathetic, transparent and participatory communication means. Strengthening continuity of care, empowering health workers and embedding community feedback mechanisms within humanitarian health responses are critical for sustaining confidence in vaccines in conflict settings.

## 1. Introduction

In contexts of humanitarian crisis and protracted conflict, the social structures and reliable information flows that communities rely on are often disrupted [1]. Daily life is impacted by fear and uncertainty, which shapes how people access, interpret and act upon information that is crucial for their health and survival. Within these fragile environments, information ecosystems, defined as the ways in which individuals interact with information [2], are altered to fit new daily realities.

At the same time, conflict brings new public health challenges and threats to communities. It is, therefore, critical to deliver public health programmes and maintain essential health services, including routine childhood vaccination. The success of vaccination programmes relies on information, but also on trust, which is defined as a relationship between individuals and systems in which one party accepts vulnerability and relies on the other’s competence and good intentions [3]. Low levels of trust in institutions, particularly towards governments and health systems, are associated with reduced confidence in vaccines [4].

The full-scale invasion of Ukraine in 2022 (see terminology note in S1 Appendix) and the country’s subsequent political and social transformation present a critical case study for understanding how trust, information ecosystems and public health interventions such as vaccination are influenced by the lived experience of crisis. In Ukraine, the urgency of this task is underscored by the persistence of approximately 15,000 ‘zero-dose’ children in Ukraine (those who have not received any routine vaccinations) and recent outbreaks of polio and measles [5,6]. Existing literature prior to the full-scale invasion highlights that vaccine confidence in Ukraine has been influenced by the country’s political history, health system performance and broader state-society relations [7,8]. Mistrust in vaccines has been linked to perceptions of corruption, weak governance and concerns about the quality and safety of state-provided services [7,9]. Compounding these domestic challenges, Russian-backed disinformation campaigns prior to the full-scale invasion weaponized health communication, using false narratives about vaccine safety and foreign pharmaceutical influence to further undermine institutional trust [10–12]. At the same time, reforms to the health system, following the 2014 Revolution of Dignity, included efforts to increase transparency, accountability and patient choice, which contributed to improvements in service delivery [8,13]. However, these gains remained uneven, with trust in vaccines continuing to reflect broader dynamics of public confidence in the state [14].

Since the full-scale invasion, several studies have explored trust in vaccination of Ukrainians displaced outside of the country. Studies in Poland and Romania detail how displaced Ukrainians tied confidence in vaccines to perceptions of institutional corruption and a historical scepticism regarding the quality and safety of state-provided vaccines [15–17]. This breakdown in trust is underscored by historical informal practices, such as the use of black markets for false certificates, and a recurrent distrust of health workers in both the home and host country [15,17]. The studies detail that while host-country settings present significant barriers, including language gaps, financial burdens, and perceived paternalism, trust can be bolstered by proactive recommendations from providers and communication that empathizes with the specific vulnerabilities of families in crisis [16,18–20].

However, much of the current literature focuses on external displacement, leaving a critical gap in our understanding of those remaining within the country. Recent evidence from frontline and Zakarpattia regions highlights that for those inside Ukraine, the crisis has introduced unique layers of complexity: internally displaced persons (IDPs) grapple with the physical loss of vaccination records and widespread fears that frequent power outages have compromised the vaccine cold chain [21]. Furthermore, while historical hesitancy persists, the psychological and financial toll of active war has impacted the ability of caregivers to manage routine health needs alongside immediate survival [21].

There remains a lack of empirical research that captures how parents experienced the conflict across different contexts including in their hometown, while internally displaced and as refugees, alongside insights from key informants involved in the public health and humanitarian response. This study therefore aims to describe the lived experiences of parents affected by the war in Ukraine, both within the country and in displacement in Poland, and how these experiences influence engagement with information and trust in vaccination, with implications for humanitarian and public health responses.

## 2. Background

The Russia-Ukraine war started in 2014, following the Revolution of Dignity which toppled President Yanukovych following months of mass protests against corruption, authoritarianism and the government’s rejection of closer ties with the European Union [22]. The initial phase involved the Russian annexation of Crimea and military action in Eastern Ukraine and was characterised by an ongoing external threat, damaged infrastructure and internal population shift in conflict affected regions [23]. It was not until the full-scale Russian invasion of Ukraine, commencing in February 2022, that the entire country was fundamentally altered [24]. This escalated the crisis, triggering one of the largest displacements in Europe since World War II, forcing millions to flee their homes within Ukraine and abroad, with a large majority of those who left the country finding refuge in Poland [25]. This mass displacement alongside the direct impact on infrastructure and daily life, placed immense strain on the remaining healthcare system and exacerbated existing vulnerabilities [26]. It has profoundly reshaped Ukrainian social and political dynamics [24].

Since the beginning of the conflict, misinformation and disinformation have been a disruptive force. Misinformation refers to the spread of unverified information that lacks a reliable evidence base, while disinformation involves the deliberate dissemination of falsehoods to mislead [27]. Prior to the full-scale invasion, Russia conducted disinformation campaigns as part of its hybrid warfare, promoting false narratives about vaccine safety and biolabs, exacerbated during the COVID-19 pandemic [28]. These campaigns contributed to pre-existing public debates about vaccination [23]. With the full-scale invasion, Russian disinformation narratives shifted away from vaccination towards justifying military aggression, delegitimising Ukraine sovereignty and undermining Western support [29]. Despite this, other rumours on vaccination persist, this includes the link between MMR and autism among others and are sometimes linked to religious views or practical concerns like vaccine storage during power outages caused by shelling [30].

Amidst these information threats, communities are active navigators of information [1]. Stuart Hall’s encoding-decoding theory argues that individuals are not passive recipients of information but interpret it through the lens of their political context, social norms and individual experiences [31,32]. This interpretation is intertwined with deeper issues of mistrust and marginalisation [4].

In Ukraine, these information processes have also played out against a backdrop of evolving vaccine sentiments. Ukraine has historically experienced low vaccination rates, with rates among the lowest in Europe before the full-scale invasion [5,33]. While there has been some progress, including since the full-scale invasion, concerning immunization gaps persist, particularly for families displaced by the war. It is against this backdrop that we seek to explore how displaced families and host communities interact with information, how their experiences shape trust in vaccines and how crisis itself reconfigures the relationship between information, trust and decision making.

## 3. Methods

### 3.1 Ethics statement

Ethics approval was obtained from the London School of Hygiene & Tropical Medicine Observational Research Ethics Committee (ref: 29965), The Institutional Review Board #1 of the Ukrainian Institute of Public Health Policy (ID: 2024-015-01), and the Bioethics Committee of the Institute of Mother and Child in Poland (Resolution: No. 62/2024).

### 3.2 Study design

This study uses a qualitative design drawing on semi-structured interviews with parents conducted in Ukraine and Poland, and key-informant interviews with journalists, academics, humanitarian responders and civil society in Ukraine and Poland and with representatives from regional public health bodies.

The research team included international (outsider) researchers from the London School of Hygiene and Tropical Medicine, local independent researchers in Ukraine and Poland and institutional collaboration with the Institute for Mother and Child Foundation in Poland and UNICEF in Ukraine and Poland. This layered collaboration was crucial for facilitating participant recruitment, navigating ethical considerations and providing important contextual information.

### 3.3 Study setting

Ukraine has a long history of community hesitancy around vaccines [33], which resulted in some of the lowest immunization rates in Europe prior to the 2022 invasion [33,34]. This legacy is often framed as a ‘crisis of governance’ characterized by a breakdown of trust in state health authorities due to perceived corruption and safety concerns [16,34]. Furthermore, anti-vaccination movements have been active in the country since the early 2000s [34]. Since the full scale invasion in 2022 there have been outbreaks of polio and measles in parts of Ukraine [35].

The full-scale invasion has had far-reaching impacts, including on the health system and access to healthcare. As of 19 February 2026, over 5.9 million refugees from Ukraine have been recorded globally, with over 5.3 million displaced Ukrainians recorded in Europe alone, three quarters of whom are women and children [36]. 3.7 million Ukrainians are internally displaced [37], and an estimated 4.1 million people are in need of urgent humanitarian assistance [38].

This study explores the experiences of families in two city settings: Lviv, Ukraine and Warsaw, Poland. This facilitated the documentation of experiences of three groups of parents, including those navigating internal displacement, displacement outside of the country and those who have not been displaced but were grappling with the impact of ongoing war in their home city. Three groups of parents were identified; (1) parents displaced to Lviv from other parts of Ukraine (2) those already residing in Lviv prior to the full-scale invasion and (3) parents who had left Ukraine and temporarily settled in Warsaw since the full-scale invasion. Internally displaced families were largely from Eastern and Southern Ukraine, regions most heavily impacted by fighting since 2014. Ukraine is a bilingual country, with Ukrainian more common in the west and Russian in the east, through as Shore [22] argues such distinctions obscure the everyday reality of casual bilingualism.

### 3.4 Participant recruitment and consent

Purposive sampling and snowballing were used to identify and recruit participants for both semi-structured and key informant interviews. Initial recruitment of families displaced to Lviv was done through temporary accommodation shelters with access facilitated by non-governmental organisations (NGO) providing services to displaced families. Parents residing in Lviv were recruited in community spaces around the temporary facilities including church, activity centres for children and online through Facebook community groups for local parents. In Warsaw, parents were reached through services for Ukrainian parents displaced to Poland, including child friendly spaces and a Ukrainian kindergarten. In all locations, initial participants referred others through snowball sampling. Participants were recruited based on their lived experience of conflict and displacement, consistent with the qualitative aims of the study.

Key informants were purposefully sampled to generate a range of perspectives including representatives from the Ukrainian government (national and local), the humanitarian response sector, the media, academia and the health sector.

In the setting of an ongoing war, the recruitment process had to remain flexible and opportunistic, shaped by rapidly changing conditions on the ground and the safety, availability and comfort of participants. Informed written consent was obtained from all participants. For interviews conducted online, participants were provided with a digital copy of the consent form in advance, which they signed and returned electronically prior to participation. This process was led by local members of the research team, who helped navigate potential power imbalance and mistrust of outsiders and authorities. A dynamic consent approach was used, allowing participants to revisit their willingness to participate during and after the interview process.

### 3.5 Data collection

Interview topic guides were informed by existing literature on trust, information and vaccine confidence in crisis settings [1] and were refined through informal discussions with a small number (approximately five) of humanitarian practitioners engaged in the Ukraine response during the fieldwork planning phase. Topic guides were iterative, evolving with the collection of data and interviews were conducted in a semi-structured manner, allowing questions to be adapted to participants’ experiences and emerging themes.

Data were collected between November 2024 and March 2025, including through in person fieldwork in Ukraine (November-December 2024) and Poland (February-March 2025). Participant recruitment for the Ukraine study site took place from 20/11/2024 to 15/01/2025, and for the Poland study-site from 10/02/2025 to 01/03/2025. Recruitment of parents occurred only during the in-country fieldwork periods at each site. Participants were eligible if they were parents affected by the conflict in Ukraine (either residing in Lviv, internally displaced, or displaced to Poland) and were willing to participate in the study. No formal exclusion criteria were applied beyond the inability to provide informed consent. Key informant recruitment continued remotely across the full recruitment windows for both sites.

Semi-structured interviews were conducted in person by the lead author and two research assistants who also acted as interpreters. Research assistants were bilingual in Ukrainian and Russian (and Polish in the Poland study site), with experience working with researchers and as journalists and interpreters. They supported recruitment, translation and cultural interpretation during interviews. Their linguistic and cultural familiarity with participants facilitated rapport, while the involvement of two interviewers enabled reflection on potential biases in data collection and interpretation. Interviews with Ukrainian parents were primarily conducted in person, with a small number conducted via Zoom where in-person interviews were not feasible. In-person interviews took place in locations convenient and familiar to participants, including homes, temporary accommodation, cafes and community spaces such as clinics or local organisations. Key informant interviews were conducted either in person or via Zoom by the lead author, with support from research assistants acting as interpreters where required.

A reflexive field diary was maintained as a tool to navigate the research process, capturing contextual insights, methodological decisions and the research team’s reflections on their positionality and interactions with participants. This approach aligns with established qualitative research practice, where reflexive writing is used to examine how researchers’ subjectivity and context influence the research process [39]. This diary documented ethical considerations, evolving dynamics in the field, particularly in Ukraine where the security situation was fluid, and aimed to contribute to transparency in the study. It served as a means for self-reflection, helping the researchers process challenges encountered during data collection and analysis.

### 3.6 Data management and analysis

Interviews were audio recorded and transcribed in the language in which they were conducted (English, Ukrainian or Russian) and then translated into English by a third research assistant. They were all assigned identification numbers and anonymised, identifiable information was redacted from the transcripts. Only co-authors had access to the data.

Data analysis commenced during fieldwork through debriefs conducted following each interview between HD, KG and HY. HD recorded these reflections in the fieldwork diary. A thematic analysis [40] was undertaken to explore themes and patterns across the datasets. This approach was selected for its flexibility and its capacity to generate a rich, contextual analysis of patterns across different qualitative datasets, particularly suited to this study given the inclusion of multiple participant groups, including non-displaced parents, internally displaced parents, parents displaced to Poland, and key informants [41]. Coding was conducted iteratively, moving between the data and emerging interpretations, and was primarily inductive, while also informed by key concepts relating to the study objectives including (i) the information ecosystem (ii) experiences of conflict and (iii) the public health and humanitarian response. These key concepts informed the development of themes and sub-themes. Coding and theme development were led by the first author, with ongoing reflection supported by fieldwork debriefs, the reflexive diary, and an iterative writing process involving the research team, including feedback on emerging interpretations through draft writing and discussion. The analysis was informed by a reflexive lens that incorporated the perspectives of both outsiders (UK-based researchers) and Ukrainian, Polish and Belarussian researchers.

## 4. Results

The characteristics of the participants are outlined in S1 Table. In total, 30 Ukrainian parents were recruited for semi-structured interviews: 12 IDPs in Lviv, 8 residents in Lviv prior to 2022 and 10 displaced to Warsaw; all but two were female. 21 key informant interviews were conducted. All interviews lasted between 45 minutes and 1.5 hours.

Based on the analysis of the data, three key themes were identified: (1) the information ecosystem in action; (2) experiences of war and displacement and its influence on trust; and (3) approaches to building and sustaining trust in vaccination.

### 4.1 The information ecosystem in action

**Fig 1:**
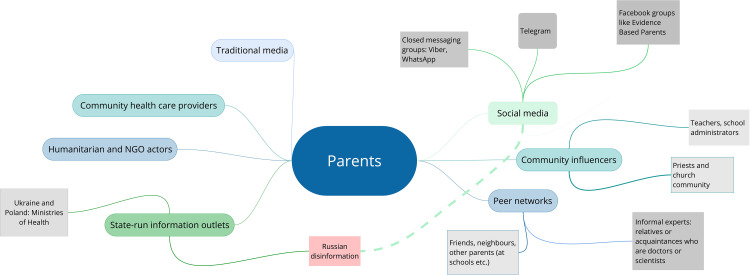
The information ecosystem mapping the diverse web of formal and informal information sources drawn on by parents and articulated in interviews. Please note some of the information sources were more relevant to different groups of parents included in the study, this is discussed in more detail below.

This theme encompasses how communities impacted by the war in Ukraine interacted with information, focusing on the sources they used, the channels through which they received information and the dynamics of information sharing and validation (see Fig 1). It highlights the active role that individuals take in seeking, sharing and interpreting information within their specific contexts.

#### 4.1.1 Key sources of information.

Parents in Ukraine drew upon a diverse range of information sources to navigate the realities of life amid protracted conflict. Digital channels, particularly Telegram, have become increasingly prominent since the full-scale invasion. Participants described the appeal of Telegram lying in the perceived authenticity and personal connection offered by individuals managing Telegram channels. Alerts provided on channels about military activity and air alerts, for instance, were often quicker and more specific about threats than the official Ukraine Air Alert App that the government recommended everyone have installed on their phones. This was particularly the case during the early days after the full-scale invasion. The system of information sharing via Telegram has matured as a tool in the years since and complements official sources such as the channels of government officials and traditional news media outlets. The following quote from KII12 demonstrates the multifaceted complexity of the information ecosystem in Ukraine, and highlights how personal or emotional connections are important predictors for which source of information is trusted:

“...*the majority receives [political] information from Telegram channels or YouTube experts. The same is about medicine, unfortunately.*..*When we ask the question, “Do you trust information?”… they explain, “I see a real person behind this Telegram channel.” For example, this is a military guy or real doctor... That is why I believe this information. It’s a problem because to combat this information, it’s very, very hard.” – [KII 12]*

Personal networks, encompassing friends, relatives and acquaintances, constituted another crucial source, with individuals often consulting these networks to validate information. When it comes to information about healthcare and vaccination, individuals employed various strategies to assess the credibility and reliability of information, cross-referencing information with multiple sources. Healthcare providers were regarded as highly trustworthy sources, particularly when they engaged in open communication and address patient concerns. Social media platforms such as Facebook served as another primary source with groups like “Evidence-Based Parents” [42] which is run by medical professionals in Ukraine and aims to facilitate access to accurate information and connections with trusted healthcare professionals as described by one parent who was a resident in Lviv prior to 2022:

*“[As for vaccinations, I trust] mainly this vaccination group on Facebook. And also, my paediatrician. I see our national vaccination calendar. I see when the recommended vaccines need to be given. I see if the vaccines are available. I take the child for a check-up to the doctor. We sign a written consent; we vaccinate without any problems. If I have any doubts… I can always get advice on Facebook, and the moderators there always answer me very well.” – [Parent 15]*

In Poland, digital tools like translation and AI-powered tools played a crucial role in bridging language gaps when accessing essential services, including healthcare and vaccination information. Google Translate was specifically used for communication with doctors who may not speak Ukrainian or English, sometimes necessitating in-person visits simply to use the translation tool, because telephone conversations were difficult as noted by one parent who has been displaced to Warsaw since 2022:

*“At some point, of course, the children got sick. My friends weren’t helping me as much because they were tired of helping everyone, and I went to the doctor by myself. I asked where is the hospital, and I went there … I somehow made an appointment with the doctors in broken English and Ukrainian. The doctor only spoke Polish. And you’re sitting there with Google Translate.” – [Parent 21]*

ChatGPT was described as more recently becoming a valuable tool, acting as a “best friend” (Parent 26) for handling bureaucracy. Participants were aware that it was imperfect, particularly for general information. Importantly, parents demonstrated not only scientific literacy but also an awareness that today’s challenge lie in navigating the coexistence of reliable and misleading information. As one parent in Warsaw explained:

*“Nowadays you can have all the information you want… probably, it’s the same in terms of vaccines and everything else, if you want to find bad information, you will find a bunch of articles, and ChatGPT will tell you about it. But if you want to know about the research, all [this] clinical evidence, you will find them too. Just ask the question.” – [Parent 21]*

Individuals employed various strategies to assess the credibility and reliability of information. This included cross referencing information with multiple sources, including healthcare providers. Some key-informants suggested that people may overestimate their ability to distinguish between high- and low-quality information.

#### 4.1.2 Trust, mis/disinformation and the reshaping of information in crisis.

Misinformation and disinformation were described as disruptive within the information ecosystem, particularly regarding healthcare. Before the full-scale invasion, Russian-backed disinformation campaigns were a consistent feature of Ukraine’s information landscape, forming part of wider hybrid warfare tactics aimed at undermining public institutions and Western-aligned health partnerships. A key-informant described that these early campaigns often focused on vaccine safety and foreign pharmaceutical influence, feeding into existing concerns about corruption and political interference in health:

*“There has been a very large Russian influence in Ukraine in general all these years, particularly in the information field. And this has always somehow concerned vaccination issues. For example, if we compare past years and this one, the same people who called themselves experts, activists, lawyers and they were against vaccination, these same people, after the start of the full-scale invasion, tried to disrupt the [military/war effort] mobilization in Ukraine.” – [KII 3]*

After the full-scale invasion, participants described how individuals and groups previously involved in anti-vaccination narratives redirected their messaging toward the war effort, including attempts to disrupt mobilisation. This shift showed that disinformation did not disappear but adapted to the conditions of escalating conflict.

Trust in traditional media was reported to be declining. Several participants described major television outlets as politically influence or connected to oligarchic ownership. As a result, people became increasingly cautious about official information and attentive to political influence when evaluating health messages.

Participants also noted that health and vaccine communication was intertwined with political narratives, a theme explored further in our companion paper on the history of vaccine politics in Ukraine [23]. Messages about vaccines were used within broader pro-Russian information efforts, framing Western vaccines as unsafe and Russian ones as trustworthy. As a key informant articulates, this reinforced the view that vaccination was not purely a medical issue but also a political one, and therefore potentially risky:

*“There was a lot of pro-Russian disinformation, and then there were still strong pro-Russian media in Ukraine. Several large television groups that promoted disinformation about Western vaccines like Pfizer and instead claimed that the Russian Sputnik was the only vaccine that works. These were pro-Russian media that played in Russian interests using Russian money. They tried to prove that Ukraine should be friends with Russia and receive a Russian vaccine from it. And the West gives a bad vaccine that will not work.*

*This message was not very popular among the people. There was no such thing as people going around and saying: give us the Russian vaccine. But as a result, it undermined trust in the very idea of vaccination, and people thought of getting vaccinated against coronavirus as a risky and dangerous event.”* – [KII 4]

### 4.2 Experience of conflict and its influence on trust

#### 4.2.1 Shapeshifting vaccine sentiments in the face of physical threats.

Sentiments around vaccination have evolved since the early days of the full-scale invasion. Attacks on infrastructure led to some questions about the safety of vaccines, primarily due to power outages. Parents reported feeling worried about maintenance of the cold chain.

*“We also had the power outage, I worried about the quality of the vaccine, whether the pharmacy or clinic kept it in proper condition, whether it was kept cold, whether it did not go bad.” – [Parent 15]*

Conversely, the war also prompted some individuals to become more proactive about vaccination to protect their families in uncertain times. Some parents, previously sceptical about vaccination, brought their children for vaccination for the first time, worried about the risk of viruses in an unstable environment. People began to place a different value on life and healthcare, seeking more guarantees.

*“At the very beginning of the full-scale invasion I made the final decision to vaccinate my children… I understood that if the situation worsened, then in fact the risk of getting sick would simply become enormous, especially here we were talking about hepatitis B and other things related to blood transfusion. Because I had no confidence that even if help was provided, that the conditions would allow protecting my children from these diseases.” – [Parent 13]*

For others, displacement and the stress of conflict could lead to vaccination falling down the list of priorities. The experiences of displaced families varied, with some prioritising basic needs over vaccination. Vaccinations were not avoided because parents were against them, but because of the uncertainty of living day-to-day as described by a key informant in the public sector:

*“Well, let’s say that during this period [during the war] the priority for all of us, and not only for children, is to preserve our own lives. We are really suffering. The whole country and every Ukrainian, both small and adult, is suffering from missile strikes, from damage to power grids, from a full-scale invasion. This is what a war does. It is a priority to protect yourself and protect others, to save your life.” – [KII 6]*

#### 4.2.2 The experience of displacement.

Displacement significantly affected access to healthcare and vaccination services, creating additional challenges for both displaced populations and healthcare providers. Initially the Ukrainian government adapted the health policy to allow displaced people to access healthcare from any doctor in the country without an official “declaration”. This declaration is a formal document signed with a patient’s chosen family doctor [43].

Parents reported linguistic, practical and psychological barriers, which not only limited access to services but also influenced the willingness and/or ability to vaccinate. People displaced from eastern regions to Lviv described tension around speaking Russian, their primary language in an environment where Ukrainian is dominant. Such experiences of exclusion or uncertainty sometimes deepened hesitation or delay, even among parents who otherwise supported vaccination. One parent displaced to Lviv from eastern Ukraine:

*“Here’s the situation regarding healthcare and the Russian language. Here on the first floor lives a woman, she’s 82 years old, she’s spoken Russian all her life, she seems to be from Popasna and there’s no place to live there at all. It’s like Chernobyl, I saw drone footage. And her blood pressure was 200. They called a doctor and doctors started... I’m sorry, when they started asking an 82-year-old woman with blood pressure over 200 why doesn’t she speak Ukrainian. And these were doctors.”-* [Parent 9]

The prioritisation of basic needs was regularly emphasized by both displaced parents and in key-informant interviews. Access to shelter, food and employment were urgent and took precedence over preventative healthcare such as vaccination.

*“I have to say that when we arrived in Lviv, it didn’t matter whether we came to Lviv or to some other place. Safety was the priority, and we were safe. All my thoughts were only about one thing. Well, imagine that you live in a house. You have everything, you are happy with everything. And in one day you just lose everything: your job, your house, and all your belongings. You came to a new place and... Well, you have nothing at all and there is little money, because I didn’t save up much. And the first thoughts were: how to live on? Where to live? What to live for? Where to get money for food? How to get a job? Those were [my thoughts]. Medical care was not needed at that time.”- [Parent 11]*

Among displaced parents in Poland, structural and financial barriers also affected access to vaccination. Differences between Polish and Ukrainian immunization schedules, together with unfamiliar administrative processes, created uncertainty for both parents and health workers. A key informant in Poland described how some healthcare providers appeared to favour treating Polish patients over displaced Ukrainian mothers, even when Ukrainian parents could communicate in Polish:

*“You have a mother coming [to Poland] … they don’t know the vaccination calendar…and it creates barriers also for the healthcare providers. Even if they* [42*] can speak Polish, it’s preferable for them [healthcare providers] to treat …Polish mothers.” [KII 21]*

In addition, among parents displaced to Warsaw, several described having to pay out of pocket for certain combinations of childhood vaccines, with costs reaching up to 700 zloty (approximately $193 USD) per dose, creating a financial barrier to access.

#### 4.2.3 Conflict, vaccine supplies and access.

The war has led to some reported vaccine supply shortages in Ukraine. Vaccines have stopped reaching some areas, with a continuous supply replaced by intermittent deliveries that do not meet needs. Over 2,000 medical facilities have been destroyed [44]. In areas of active fighting, it is difficult to reach a medical facility [45]. A national-level key informant described the impact of these disruptions:

*“We definitely had such problems [with vaccine supplies], especially after I remember the first massive missile attack on Ukraine in October [2022], which was particularly serious. And it was a problem to find an international carrier that could deliver [vaccines] to Ukraine. Because logistics companies, drivers from other countries, simply refused to enter our country.” – [KII 7]*

Power outages and attacks on infrastructure have accelerated activities to update the cold chain system (that began during the COVID-19 pandemic) [KII 10]. Key informants described how the Centre for Public Health, in partnership with humanitarian organisations like UNICEF and the World Health Organization with support from the World Bank, developed a system for preserving vaccines in conditions of frequent and unpredictable power outages [KII 7; KII 10]. At the regional and facility level, cold storage and transport systems were modernised to strengthen vaccine storage and distribution [KII 8; KII 5].

However, as another key-informant noted, these efforts did not fully eliminate inequities in access. While regional hubs benefitted from upgraded infrastructure, key informants noted that remote and frontline communities continued to face challenges in vaccine delivery, health worker shortages and disrupted data systems [KII 7; KII 8; KII 10]. The movement of populations across Ukraine and outside the country further complicated vaccine management and monitoring [KII 6; KII 7; KII 8; KII10].

*“If you, for example, have a war and you have people fleeing out of the country or moving around the country, how can you measure, for example, what is your target audience if you don’t have the objective data?”- [KII 10]*

### 4.3 Approaches to building and sustaining trust in vaccination

This section describes how trust in vaccination was both perceived and built. Parents identified the forms of communication and interaction they found most trustworthy, while health workers and public health actors outlined the strategies, they used to foster that trust.

#### 4.3.1 Communications tactics.

Parents described relying on communications channels that felt interactive, responsive and personal. Being able to ask questions and receive timely, individual responses was seen as a key advantage of digital spaces. As one key informant explained, online groups run by professionals offered an accessible and trusted space for discussion:

*“In Ukraine there is actually a very cool Facebook group about vaccination, which is run by professional experts. And there you can ask any questions, that [is] growing very fast, and it actually helps hundreds of thousands of parents to make the right decisions.” – [KII 3]*

This sense of dialogue and proximity helped build credibility when responses came from identifiable health professionals or locally embedded moderators, and when timely updates addressed concrete worries (for example schedules and availability.) However, the same digital spaces also hosted misinformation and disinformation, underscoring the need for moderation and verification of health experts.

Health workers similarly emphasized their belief in the need for clear, empathetic communication and for sharing personal experiences to build credibility during consultations. Many described official or verified social-media accounts to help parents cross-check information, strengthening trust through transparency and responsiveness.

At the institutional level, efforts were made to embed transparency and reliability across the health system’s communications structures. The launch of Ukraine’s National Immunization Portal [46], provided a centralised, evidence based resource for both the public and healthcare professionals. At the same time one key-informant described efforts by the Ministry of Health to build transparency and therefore trust:

*“The State Expert Centre [of the Ministry of Health] …track all safety issues… they openly respond. You can go to their website and observe the number of adverse events [related to vaccination] based on their types and severity, it’s open information… this transparency that was built is one of the key [factors in trust.]” [KII 9]*

Together these efforts show that effective communication relies on responsiveness, clarity and empathy. These same themes were echoed in accounts of community engagement and outreach activities described below.

#### 4.3.2 Community engagement.

Humanitarian responders reported the importance of working through trusted local figures to strengthen vaccination outreach. One key informant described how a family doctor worked between the regional authorities and a local priest in a village with low vaccination rates to build trust and increase vaccination uptake, and went on to explain how such partnerships were typically established:

*“We realize that it’s really crucial to be in touch with local leaders, and some local groups … It can be the head of the village [or] director of a school. It can be some people from … religious groups… First of all, we communicate with representative of regional CDC [Centre of Disease Control], then with head of the village. Then, we try to figure out how we can proceed, and who can be our focal point.”- [KII 9]*

Key informants also described how civil society organisations helped build networks of health workers across the country, providing training and knowledge exchange to strengthen their role as vaccination advocates. These networks were reported to enhance coordination between healthcare professionals and local authorities, particularly in rural areas. Local authorities described examples of collective action, including a vaccination congress that brought together health, education and military representatives, as well as parents to discuss vaccination.

#### 4.3.3 The critical role of health workers.

Parents most frequently described their primary healthcare doctors as the most trusted source of vaccination information. Positive experiences, clear communication and continuity of care were seen as key to building trust, while negative encounters, such as perceived neglect, limited knowledge or paternalistic behaviour, undermined confidence. One parent illustrated how personal interactions with healthcare providers directly shaped her sense of trust:

*“When my son was just born, I didn’t really [trust] the doctor because she forgot to remind me [about the vaccine]. I just looked into the calendar and saw that we had to do something, but no one told me anything, so I checked it myself. We called the nurse and she* [47*]: yes, you need to come. And I asked: is there a vaccine or not? It was with that paediatrician [that I had a problem]. When I changed doctors, such situations did not arise. Well, yes [in Lviv I trust the paediatrician].” – [Parent 11]*

Key informants explained that the post-2014 health reform, which emphasised the role of family doctors, helped foster these more trusted and patient-centred relationships. Family doctors were often the first point of contact with the health system, and their active engagement was viewed as critical to sustaining vaccine uptake. One key-informant reflected on how the reform strengthened both the agency of health care workers and the confidence of patients:

*“I would say that health reform worked for the vaccines more because the responsibility of the primary healthcare physician is a little bit more. The relationship with the patient is [closer] because they want to be with this doctor, so everything depends on the doctor and on the nurse, on this team. If they are proactive towards vaccination, the reform gave them more power to them and more trust to the patient.” – [KII 10]*

Taken together, these findings illustrated how conflict has reshaped information, trust and decision making around vaccination, and how families, health workers and institutions have adapted within these constraints.

## 5. Discussion

The findings of this study showed that conflict does not simply interrupt vaccination programmes but reconfigures trust in vaccination and interactions with the information ecosystem. This aligns with broader literature from other conflict and crisis affected settings, which highlights how experiences of insecurity, displacement and political instability disrupt established trust processes [1]. While also reflecting the interactions between mistrust and vaccination whereby confidence in vaccines is contingent upon trust not only in the product itself but also in the providers, the health system, government and wider information environment that underpin vaccination decisions [3,16]. The findings point to three interrelated ways in which crisis reshaped vaccines attitudes: (i) by disrupting access and continuity of care, (ii) by vaccination becoming a means to assert agency amid uncertainty and (iii) by reconfiguring the way parents interacted with information.

### 5.1 Disrupted access and continuity of care

In line with wider evidence [1], this study also found that access and vaccine confidence were mutually reinforcing. We found that conflict and displacement disrupted health-seeking, with vaccination often deprioritised as families navigated documentation, housing and immediate safety. Language barriers added to these challenges, as did experiences of tension for Russian-speaking IDPs in Lviv and the need to interact with Polish health services abroad. Similar dynamics have been documented in other crisis contexts, where physical, financial and linguistic barriers reduce both access and confidence in vaccines. Studies from Haiti, Somalia and the Democratic Republic of Congo showed that when services were fragmented or perceived inequitable, convenience barriers become entwined with mistrust of institutions [48–50]. Conversely, research from Gaza, Sierra Leone and South Sudan has shown that when humanitarian actors strengthened continuity of care through community-based services, trust in vaccination can improve despite instability [47,51,52]. These findings underscore that access is not a purely logistical concern but a relational one, where ease of obtaining vaccines can influence people’s willingness to vaccinate. Within the WHO SAGE definition of vaccine hesitancy, “convenience” is recognised as one of the key behavioural drivers of vaccine uptake [53]. In fragile or disrupted settings, reduced (or improved) convenience can reshape vaccine attitudes and trust in the system providing them.

### 5.2 Vaccination as a means to assert agency amid uncertainty

At the same time, some parents described an initial increase in vaccination in the early days of the invasion driven by a desire to protect their children and assert agency. This sense of agency, despite instability, resonates with Henrik Vigh’s concept of *crisis as chronicity* [54] which frames crisis not as temporary rupture but as an ongoing social condition through which people make meaning and navigate daily life. Within this framing, vaccination became more than a biomedical act it was a moral and protective one, symbolising an effort to preserve normality and care for one’s family in the midst of disruption. The results showed that even amid profound uncertainty, community resilience and mutual support persisted, parents mobilised personal and social networks to find information about vaccines, locate supplies and navigate administrative barriers. These behaviours reflect how health decision making during conflict is shaped by the interplay of structural constraints and active meaning making. Recognising these parents as rational health citizens, demonstrating political agency through their health choices allows us to resist the interpretive marginalisation often imposed by a ‘foreign gaze,’ which flattens such survival strategies into labels of simple hesitancy or ignorance [47,55–57].

Similar patterns have been documented in other humanitarian settings. During the Ebola crisis in Sierra Leone, for instance, vaccination and participation in biomedical research were interpreted, by some, as moral acts of protection for the collective good, even amid pervasive mistrust [51]. In Beckmann’s [58] work on AIDS treatment in Tanzania, therapeutic engagement was shown to provide a means of reasserting social value and agency within chronic crisis. Likewise, research on community led-ethnographic work in Sierra Leone, demonstrated that when people were meaningfully involved in decision making, vaccination became embedded in local notions of responsibility and survival [51]. Recent evidence from the polio vaccination campaign in Gaza in 2024 similarly challenges depictions of people in conflict as passive recipients of aid. Despite destroyed infrastructure, logistical challenges and insecurity high vaccination coverage was achieved [59]. Amid crisis, individuals actively engage with health systems, making informed decisions grounded in lived experience and shifting perceptions of risk [60].

### 5.3 The changing role of information

Information is a critical part of the shifting interactions between trust and vaccination. As Larson [61] argues, the “trust chain” is often the more important lever of vaccine acceptance than any individual piece of information. However, without layers of confidence, even the most scientifically sound and well-communicated information may fail to persuade. This underscores that trust and information are interdependent; information is filtered through prior beliefs, social norms and lived experiences that determine whether it is received as credible or not. Crisis contexts amplify these dynamics. Under conditions of fear and uncertainty, people’s assessments of information are shaped by proximity, authenticity and perceived care, rather than by information alone [51,62].

Our findings showed that parents described deeper interactions with digital channels, particularly Telegram, where information was valued for its immediacy and perceived authenticity. Unlike traditional media, which was often perceived as politicised or slow, telegram channels and smaller online groups were trusted for providing rapid, direct and emotionally resonant updates. Hall’s encoding/decoding model [31] helps illuminate this process: audiences are not passive recipients of messages but interpret information through dominant, negotiated or oppositional readings based on their lived realities. Parents’ accounts revealed how their readings of vaccine-related information were mediated by personal experiences. While Russian disinformation campaigns had tried to exploit existing fractures and mistrust, participants also linked scepticism to historical corruption and social connections to shaping attitudes [23]. In line with Hall’s framework, the findings show that even factually inaccurate information was not always rejected outright. It was interpreted through individual and collective histories of trust, displacement and uncertainty.

In this shifting information landscape, key informants highlighted the need for tailored and transparent communications strategies that resonated with people’s lived experience. Approaches that combined clarity, empathy and two-way dialogue were viewed as most effective, reflecting parental preference for communication spaces where they could ask questions, receive timely responses and see evidence of accountability. This aligns with emerging literature that moves away from focusing on information in isolation to fostering communication in transparent, accountable and trusted relationships [63].

### 5.4 Strengths and limitations

We acknowledge several limitations that could impact the findings of this research. Given the study demonstrates the importance of context on information ecosystems, trust and vaccine sentiments, the focus on Ukrainian parents impacts transferability to other populations. The study was based on a relatively small, context-specific sample, and while this is appropriate for in-depth qualitative inquiry, the findings are not intended to be statistically generalisable to all Ukrainian populations, including those in other regions of Poland or beyond. We also could not investigate specific narratives around individual vaccines in depth (e.g., MMR, polio), instead providing an overview of the complex factors shaping vaccine confidence in a fast-changing social context. Participant recruitment relied in part on snowball sampling, which was appropriate for accessing participants in a conflict-affected setting but may have influenced the composition of the sample and the perspectives captured. The predominance of mothers in the sample is also a limitation, as it may reflect gendered caregiving roles and influence the perspectives captured, with fathers underrepresented, in part reflecting the context of the conflict, where men of military age were generally unable to leave Ukraine while women more commonly relocated with children. We also acknowledge that time constraints, in part because of the security situation in Ukraine at the time of data collection, may have contributed to a lack of depth.

Authors in the team from outside of Ukraine and Poland acknowledge our own biases as outsiders and the privilege of working from settings not directly impacted by the reality of war. We have strived to sensitively navigate power dynamics and language barriers through reflexivity in our analysis. We also recognise that this research was conducted within a global health landscape often shaped by a ‘foreign gaze’, where researchers from international institutions may be afforded greater credibility than local experts [55].

Ukraine is a country that carries deep scars of colonialism, and as part of our efforts to contribute to more equitable and contextually grounded research, the study was conducted by a team with diverse and complementary perspectives, including researchers and practitioners from Ukraine and Poland. This composition was a deliberate methodological choice to move beyond extractive models of international research and to ensure that researchers from the study contexts were meaningfully involved in the scientific and interpretive dimensions of the work [55,64]. These collaborations occurred at every stage, from study development to fieldwork planning to thematic interpretation. We sought to reduce the risk of misrepresentation and ensure that findings remained grounded in local realities.

Our local colleagues occupied the fluid status of ‘in-betweeners’: they possessed the local status required for privileged access and empathetic understanding of the community’s survival priorities, yet they were also positioned as ‘outsiders’ through their roles in this academic project [65]. This positioning enabled the team to balance proximity to participant’s lived experiences of war with the analytical distance required for qualitative research.

We view our commitment to shared authorship and collaborative analysis not as a search for moral innocence, but as a continuous practice of epistemic accountability [64]. By adopting this critically embedded approach, we strive to ensure that participant narratives are not flattened into operational data but remain recognized as valid political subjectivities [56,64].

We also acknowledge that this study has notable strengths. The design enabled cross-border research with displaced populations, documenting experiences of parents still in Ukraine and those now in Poland. This is relatively rare in crisis research and programming and provided a unique opportunity to identify both shared experiences and key differences in how conflict and displacement shaped interactions with information and vaccine decisions. While our analysis highlights the overall information ecosystem, we recognise that there are important sub-systems within it, such as the role of Russian-speaking communities, that warrant further research to inform more targeted, audience centred communications strategies.

### 5.5 Recommendations for practice

Trust in public health programmes, including vaccination is shaped by the interplay of historical, political and social factors. In Ukraine, mistrust stems from past failures in vaccine procurement, systematic corruption and historical injustices. More than a decade of war has added another layer of disruption, including through disinformation campaigns and the more recent country-wide impact of the full-scale invasion. Addressing the challenges requires a system-wide approach that prioritises transparency in vaccine management, from procurement and supply chains to safety monitoring. The State Expert Centre’s transparent pharmacovigilance reporting in Ukraine, for example, provides a potential model for other conflict-affected settings, demonstrating how openness around vaccine safety can contribute to rebuilding institutional trust.

Our findings indicate several practical measures to improve vaccination uptake in conflict-affected communities. Healthcare workers are central to building trust in the health system and should be supported through strengthened health systems and community health structures. Building on previous health reforms, undertaken by acting Minister for Health, Ulana Suprun, which emphasized the role of family doctors, could improve trust by raising regard for this profession and fostering closer relationships [43].

Trust building extends beyond the health system. Understanding communities’ daily realities, listening to concerns and integrating local practices into health offerings are important. This requires audience-centred communications strategies that operate within the existing information ecosystems. Such approaches are already being implemented by humanitarian responders, for example community feedback mechanisms and rapid qualitative assessments, and evidence shows they are feasible even during conflict [66,67]. At the same time, our findings suggest that humanitarian actors will increasingly need to engage with the growing use of AI tools such as ChatGPT, as individuals draw on them to navigate linguistic and bureaucratic barriers, raising both opportunities and challenges for accuracy and trust.

Finally, communications strategies need to be tailored to the specific features of crisis-affected contexts. There is no one-size-fits-all approach; strategies must account for the political, social and historical factors that influence trust in each setting, while addressing structure barriers to accessing vaccines [1].

### 5.6 Recommendations for research

Further research is needed to understand the complex interplay of information, trust and healthcare in other conflict settings. The importance of documenting how communities impacted by conflict interact with information ecosystems goes beyond the realm of vaccines; it is critical for understanding social cohesion, political structures and providing an evidence base for the recovery agenda of conflict affected countries.

Investigating the evolution of digital channels and the trust individuals place in them needs examination, including how experiences of war, displacement and previous interactions with health systems influence perceptions of risk associated with vaccinations. Documenting strategies used by humanitarian actors and public health responders to build trust and promote vaccination will be helpful for informing further interventions. Conducting longitudinal studies to assess the long-term impact of health reforms and interventions on vaccination rates and public health outcomes, as well as the effectiveness of more targeted, personalised social mobilisation strategies, would provide valuable insights as the situation in Ukraine continues to evolve.

## 6. Conclusion

This research demonstrates the complex interplay of trust, information and confidence in vaccines in the context of protracted conflict in Ukraine. The findings affirm that individuals are not passive recipients of information, but rather active navigators, interpreting and engaging with diverse information streams, both online and offline, through the lens of their cultural beliefs, social norms and individual experiences. These processes are deeply tied to (mis)trust and marginalisation but also shifting terrains, heavily influenced by social and cultural contexts and historical factors.

The full-scale invasion has altered these dynamics through disrupted information ecosystems and shifting community priorities towards immediate survival needs. This has implications for vaccine sentiments and uptake. As the war continues and Ukraine looks to what the future might hold for its citizens, strengthening health systems, empowering health workers and implementing community-centred communications strategies are crucial for rebuilding trust and strengthening public health systems.

## Supporting information

S1 Appendix Terminology note. Explanation of terminology used in relation to the war in Ukraine and displacement status in Poland and the European Union [68].
(DOCX)

S1 Table Participant characteristics. Summary of participant characteristics and interview sample across parent, caregiver and key informant groups.
(DOCX)

S1 Text Interview topic guides. Semi-structured interview guides used with parents, caregivers and key informants, including topic guides for humanitarian response actors and broader case study contextual interviews.
(DOCX)
