# Supplementary material for: “This is what a war does”- Trust, information ecosystems and childhood vaccination among Ukrainian parents: A qualitative study
Source: PLOS Glob Public Health. 2026 Jul 2;6(7):e0006742. doi: 10.1371/journal.pgph.0006742 (PMC13327253; doi:10.1371/journal.pgph.0006742)
Supplement: S1 Appendix — Explanation of terminology used in relation to the war in Ukraine and displacement status in Poland and the European Union [68]. (DOCX) [file pgph.0006742.s001.docx]

**Terminology note:**

In this paper, we use the terminology of “full-scale invasion” to refer to Russia’s actions since February 2022 and refer to the beginning of the war as the annexation of Crimea and military action in Eastern Ukraine commencing in 2014. We also use the term displaced people as Ukrainians have not been provided with refugee status, rather temporary protected status in Poland (and the EU) [68].
