# Supplementary material for: “This is what a war does”- Trust, information ecosystems and childhood vaccination among Ukrainian parents: A qualitative study
Source: PLOS Glob Public Health. 2026 Jul 2;6(7):e0006742. doi: 10.1371/journal.pgph.0006742 (PMC13327253; doi:10.1371/journal.pgph.0006742)
Supplement: S1 Text — Semi-structured interview guides used with parents, caregivers and key informants, including topic guides for humanitarian response actors and broader case study contextual interviews. (DOCX) [file pgph.0006742.s003.docx]

**S1 Text. Interview topic guides**

### Interview guides: key informants in humanitarian response

| **Project: Understanding information ecosystems and trust, in crisis contexts, through the lens of childhood vaccination campaigns** | |
| --- | --- |
| Date |  |
| Place of interview |  |
| Interviewer |  |
| Organisation |  |
| **Interview procedure:** You are being asked to participate in a research study exploring how the experience of crisis shapes information ecosystems within childhood vaccination campaigns. During this interview you will be asked to respond to several open-ended questions. You may choose not to answer any or all of the questions. The procedure will involve taping the interview, and the tape will be transcribed verbatim. | |
| **Informed consent:** Please sign/record informed consent signalling your willingness to participate. | |
| **Questions** | |
| **Part 1: The context** | |
| Can you explain your role and the work that you do? | |
| Can you describe the operating environment for humanitarian and development response?  Probe: could you provide examples | |
| What are some of the contextual realities that are specific to working in this environment?  Probe: could you provide examples | |
| **Part 2: vaccination campaigns** | |
| What are the major challenges and opportunities for implementing effective vaccination campaigns in this setting?  Probe: could you provide examples | |
| How do you understand and navigate cultural sensitivities and contextual factors? | |
| How do different stakeholders including partners, community leaders, government representatives and the military interact in these processes?  Probe: could you provide examples | |
| What are the different elements of developing the community engagement aspects of vaccination campaigns?  Probe: could you provide examples | |
| What indicators do you use for monitoring the success of vaccination?  Probe: could you provide examples | |
| **Part 3: information programming** | |
| From your experience, what strategies have been effective in building trust between humanitarian organisations and service providers and communities in the context of vaccinations?  Probe: could you provide examples | |
| Are there specific communications tools or technologies that have proven successful in reaching under-vaccinated communities?  Probe: could you provide examples | |
| Are there specific lessons you have learned, or insights you have gained that have informed later programming?  Probe: could you provide examples | |
| Is there anything else you would like to add? | |
| **Closing:** Thank you for your participation in this interview. We appreciate you taking the time to do this. We may contact you in the future for the purpose of follow up questions. Should you need any further information please contact us directly. | |

**Interview guide: key informants about the broader case study context**

| **Project: Understanding information ecosystems and trust, in crisis contexts, through the lens of childhood vaccination campaigns** | |
| --- | --- |
| Date |  |
| Place of interview |  |
| Interviewer |  |
| Organisation |  |
| **Interview procedure:** You are being asked to participate in a research study exploring how the experience of crisis shapes information ecosystems within childhood vaccination campaigns. During this interview you will be asked to respond to several open-ended questions. You may choose not to answer any or all of the questions. The procedure will involve taping the interview, and the tape will be transcribed verbatim. | |
| **Informed consent:** Please sign/record informed consent signalling your willingness to participate. | |
| **Questions** | |
| **Part 1: Context** | |
| Can you tell me a little bit about yourself, and what you do? | |
| Can you describe the situation here, especially concerning key features of the crisis? | |
| What are the security and political dynamics?  Probe: could you provide examples | |
| What are the cultural and historical dynamics?  Probe: could you provide examples | |
| **Part 2: Community impact** | |
| How has the situation affected daily lives of individuals and communities?  Probe: could you provide examples | |
| How have communities adapted?  Probe: could you provide examples | |
| How has access to basic services (health, education etc) been affected?  Probe: could you provide examples | |
| **Part 3: Community response** | |
| From your perspective how are the humanitarian response or development partners perceived in the community? | |
| Are communities engaged in developing solutions?  Probe: could you provide examples | |
| What do you think lies ahead for communities here? | |
| Is there anyone else I should talk to as I try to understand the situation? | |
| Is there anything else you would like to tell me? | |
| **Closing:** Thank you for your participation in this interview. We appreciate you taking the time to do this. We may contact you in the future for the purpose of follow up questions. Should you need any further information please contact me directly. | |

**Interview guides: parents**

| **Project: Understanding information ecosystems and trust, in crisis contexts, through the lens of childhood vaccination campaigns** | |
| --- | --- |
| Date |  |
| Place of interview |  |
| Interviewer |  |
| Role |  |
| Organisation |  |
| Demographic info |  |
| **Interview procedure:** You are being asked to participate in a research study exploring how the experience of crisis shapes how we gather and use information including relating to vaccination campaigns. During this interview you will be asked to respond to several open-ended questions. You can choose not to answer any or all of the questions. The procedure will involve taping the interview, and the tape will be transcribed verbatim. Your results will be confidential, and you will not be identified individually. | |
| **Informed consent:** Please sign/record informed consent signalling your willingness to participate. | |
| **Questions** | |
| **Part 1: Building rapport and understanding individual experience.** | |
| Can you tell me a little bit about yourself, your family and your daily life?  Probes: where do you live/ who do you live with/ what is your daily routine | |
| Can you describe your personal experience during the last few years?  Probes: how did you end up here/ would you like to tell me about your journey/ can you describe any significant events | |
| Can you explain about your connections with members of the community during this time?  Probes: who are the most important people in your daily life? How do you support and rely on each other in the community? | |
| **Part 2: Vaccination (perceptions/attitudes)** | |
| How has the crisis affected your ability to access healthcare services, including vaccinations, for your children?  Probe: could you provide examples | |
| What do you know about vaccinating children?  Probe: How did you learn about vaccinations and health services? | |
| Can you describe your experience around vaccinating your children?  Probes: are your children vaccinated/ why did you make that decision | |
| **Part 3: Information (contextualising the assumptions of the vaccination programme)** | |
| In your community, how is information about vaccination shared and discussed?  Probes: How do people generally gather information? | |
| Are there certain individuals or groups in the community that people generally trust when it comes to vaccination information?  Probe: Are there community leaders who you turn to for your information? | |
| Can you describe the information provided by health workers who visit your community?  Probe: when do health workers visit your community and when they come what kind of information do they share? | |
| Do you feel that the information provided about vaccinations is clear and easy to understand? | |
| Is there anything else you would like to tell me? | |
| **Closing:** Thank you for your participation in this interview. We appreciate you taking the time to do this. We may contact you in the future for the purpose of follow up questions. Should you need any further information please contact me directly. | |
