# Supplementary material for: “This is what a war does”- Trust, information ecosystems and childhood vaccination among Ukrainian parents: A qualitative study
Source: PLOS Glob Public Health. 2026 Jul 2;6(7):e0006742. doi: 10.1371/journal.pgph.0006742 (PMC13327253; doi:10.1371/journal.pgph.0006742)
Supplement: Attachment — Submitted filename: Response to reviewers.docx [file pgph.0006742.s005.docx]

PLOS
Nine Hills Road
Cambridge, CB2 1GE
United Kingdom

23 April 2026

Dear Editorial team,

We thank the editor and reviewers for their constructive and thoughtful feedback on our manuscript titled: *“This is what a war does”- Trust, information ecosystems and childhood vaccination among Ukrainian parents: a qualitative study*.

We have carefully revised the manuscript in response to all comments, which have helped to strengthen the clarity, rigour and contribution of the paper. Our detailed responses are provided below, with corresponding changes reflected in the revised manuscript.

| **Reviewer 1** | | |
| --- | --- | --- |
| **Reviewer feedback** | **Response to reviewers** | **Page/s** |
| Introduction: Consider foregrounding in the Introduction the persistence of approximately 15,000 zero-dose children in Ukraine (p. 7). While this statistic is presented later in the manuscript, introducing it earlier would sharpen the public-health urgency that frames the study. | As suggested, we have foregrounded the statistic regarding the 15,000 zero-dose children in Ukraine early in the Introduction. | 3 |
| Discussion: The paper would benefit from a more explicit interrogation of positionality, including outsider privilege and colonial epistemologies (p. 25). While reflexivity is addressed, making these dimensions more central would deepen methodological transparency and align with decolonial debates in global health research. | We have expanded the positionality section to more explicitly reflect on outsider perspectives and power dynamics in global health research, and to describe how collaboration with researchers and practitioners based in Ukraine and Poland informed the analysis and interpretation of findings. | 29-30 |
| Conclusion: The findings on the pragmatic use of AI-mediated tools such as Google Translate and ChatGPT could be taken further by proposing their institutionalisation as adjunct public-health tools for displaced populations navigating bureaucratic and linguistic barriers (p. 12). | We have revised the Recommendations for Practice section to incorporate the implications and opportunities of AI tools for displaced populations. | 31 |
| Conclusion:  Re-emphasise the State Expert Centre’s transparent pharmacovigilance reporting as a cornerstone of rebuilding institutional trust, positioning it more clearly as a transferable policy model for other conflict-affected health systems (p. 19). | We have revised the Recommendations for Practice section to highlight transparent pharmacovigilance systems, including the State Expert Centre in Ukraine, as a transferable model for building institutional trust. | 31 |
| **Reviewer 2** | |  |
| **Reviewer feedback** | **Response to reviewers** | **Page/s** |
| Introduction:  1.The Authors state:  Politically, low levels of trust in institutions, particularly towards governments and health systems, are significantly associated with reduced vaccine confidence.  and then cite the only one reference (5).  This is a very important observation that needs additional references to support. I would suggest adding references that concentrate specifically on Ukrainian migrants and refugees, such as:  doi: 10.1007/s00431-024-05847-2  doi: 10.2807/1560-7917.ES.2024.29.39.2400159.  doi: 10.1186/s12889-020-10105-9. | As suggested, we have expanded the Introduction to ground the discussion of institutional trust within the specific context of Ukrainian populations, incorporating the recommended references [Hoffmann et al. (2024), Winters et al. (2024), and Ganczak et al. (2021)] to further support our observations regarding vaccine confidence among migrants and refugees. | 3-4 |
| 2.Please define the Research ‎Gaps regarding the study’s main theme. | We have revised the introduction to more clearly define the research gap, particularly the limited evidence on lived experiences of vaccination within Ukraine and across different forms of displacement. | 4-5 |
| 3.The manuscript would benefit from highlighting the study's novelty. | We have strengthened the articulation of the study’s novelty by clarifying its cross-context design and focus on information, trust, and vaccination among affected populations. | 5 |
| Methods:  The authors state that “Ukraine has a long history of community hesitancy around vaccines” and cite only one relevant reference (23). Please consider citing more articles in the same vein; it is not mandatory, but here, you will find some interesting manuscripts that you can consider citing and adding to your references.  Vaccine Hesitancy in Ukraine: The Sign of a Crisis in Governance? \| Wilson Center.  doi: 10.3390/healthcare13141744.  doi: 10.3389/fpubh.2024.1380627.  doi: 10.1186/s12889-020-10105-9.  doi: 10.3390/ijerph20010712. | By drawing on the suggested literature, we now provide a more nuanced explanation of how the 'crisis of governance' and long-standing anti-vaccination movements have shaped the pre-existing attitudes that continue to influence the participants in our study. | 3-4, 7-8 |
| They further state: As of July 2025, over 5.6 million refugees from Ukraine have been recorded globally, with nearly 5.1 million displaced Ukrainians recorded in Europe alone, three-quarters of whom are women and children (26). 3.3 million Ukrainians are internally displaced (26), and an estimated 4.8 million people remain in need of critical humanitarian assistance inside Ukraine and among Ukrainians in neighbouring countries (27).  Updating this information to the end of 2025 would be valuable. | These numbers have been updated with the latest estimates from UNHCR and UNOCHA 2026. | 8 |
| Informed written consent was obtained from all participants. How did it work with participants interviewed online? | We have clarified the consent procedures for online interviews in the Methods section. | 9 |
| Interview topic guides were informed by the related literature and consultations with humanitarian responders currently working in Ukraine.  “the related literature” – references are important to add.  “consultations with humanitarian responders currently working in Ukraine”. How many of them helped to inform the interview topic guides? | We have clarified the source informing the interview topic guides by adding a reference to our recent scoping review on trust, information and vaccine confidence in crisis settings, and specifying that approximately five humanitarian practitioners were consulted during the fieldwork planning phase, noting that these consultations were informal. | 9 |
| My inclination is that the authors did not test the interview guide before launching it. What about the pilot study? Were any amendments made after piloting? If so, please describe. A brief description of all included topic guides and their attachment in the Appendix would be valuable. | As this study used a semi-structured qualitative design, the interview guides were not formally piloted. Instead, they were iteratively refined during early interviews to ensure clarity and relevance to participants’ experiences. We have clarified this in the Methods section. | 9 |
| What were the exclusion/inclusion criteria in this study? | We have clarified the inclusion criteria for participants, noting that parents affected by the conflict in Ukraine were eligible to participate, and that no formal exclusion criteria were applied beyond the ability to provide informed consent. | 10 |
| Semi-structured interviews were conducted in person by the lead author and two research assistants who also acted as interpreters. What was their experience in conducting the semi-structured interviews with migrants? Which backgrounds did they represent? A variety of backgrounds allows for the reduction of potential desirability bias, regarding shaping of responses due to the role of the interviewer. | We have added detail on the background and role of the research assistants in the Methods section. | 10 |
| Were interviews with Ukrainian parents conducted in person or via Zoom? Where did all of the invited Ukrainian parents meet with the researchers for the interviews? | We have clarified that interviews with Ukrainian parents were primarily conducted in person, with a small number via Zoom, and provided detail on interview settings. | 10 |
| Key-informant interviews were conducted either in person or via Zoom. Who was the interviewer? | We have clarified who conducted the key informant interviews in the Methods section. | 10 |
| A reflexive field diary was maintained as a tool to navigate the research process, capturing contextual insights, methodological decisions, and the research team’s reflections on their positionality and interactions with participants. Was this a novel approach or had it been previously reported? Please specify. | We have clarified that the use of a reflexive field diary aligns with established qualitative research practice and added a supporting reference. | 11 |
| Did Ukrainian participants represent adequate proportions for age, for participants with secondary/university degrees, and for having/not having a child to sufficiently explore the study objectives? | As this is a qualitative study, the aim was not statistical representativeness; we have clarified that participants were recruited based on their lived experience of conflict and displacement in line with the study objectives. | 9 |
| Why was the thematic analysis used in the study? What are the benefits of such analysis? | We have clarified the rationale for using thematic analysis, including its suitability for analysing patterns across multiple qualitative datasets involving different participant groups and key informants. | 11 |
| What actions helped researchers to proceed with an accurate qualitative analysis? Which steps were taken by the Authors until consensus could be achieved? E.g., intensive literature searches (textbook chapters and articles on qualitative research methods – which ones?), articles on qualitative studies regarding the main themes of the study – which ones?), discussions (conducted in which way?). | We have clarified the steps taken to support rigorous qualitative analysis, including that coding and theme development were led by the first author, with ongoing reflection supported through fieldwork debriefs, reflexive documentation, and an iterative writing process involving the research team. | 12 |
| How were the data analyzed thematically?  Was the process of coding and development of themes inductive in nature, or did the authors plan beforehand to adhere to a pre-existing theoretical framework? | We have clarified that coding was conducted iteratively and followed a primarily inductive approach, while also being informed by key concepts from the literature and study objectives. | 11 |
| There is a repetition regarding the Ethics approval (pages 12 and 31). Please, modify. | The repeated statement of ethics approval has been removed from page 31. | 31 |
| Results  Figure 1: The authors analyzed “parents and caregivers”; however, according to Table 1, only parents were interviewed. Please clarify. | We have revised Figure 1 to refer consistently to “parents” in line with the study sample. | 13 |
| The quotation “Even if they [parents] can speak Polish, it’s preferable for them [ healthcare providers] to treat with Polish mothers.” is unclear. Please clarify. | We have added a clarifying line above to quotation to clarify. | 20 |
| „…several parents described having to pay out of pocket for certain vaccines, with costs reaching up to 700 zloty (approximately $193 USD) per dose…” Which parents? Ukrainian migrants in Warsaw or Ukrainians in Lviv? For which vaccines? Please clarify. | We have clarified that these experiences refer to parents displaced to Warsaw and specified that these were childhood vaccines. | 20 |
| The Centre for Public Health, in partnership with humanitarian organisations like UNICEF and the World Health Organization with support from the World Bank, developed a system for preserving vaccines in conditions of frequent and unpredictable power outages. At the regional level, cold storage and transport systems were modernised, and primary health facilities received grants for backup generators for vaccine storage. Reference is needed to support this statement. | We have clarified that this statement is based on key informant interviews. | 21 |
| While regional hubs benefitted from upgraded infrastructure, remote and frontline communities continued to face challenges in vaccine delivery, health worker shortages and disrupted data systems. The movement of populations across Ukraine and outside the country further complicated vaccine management and monitoring.  Reference is needed to support this statement. | We have clarified that this statement is based on key informant interviews. | 21 |
| While also reflecting the interactions between mistrust and vaccination whereby confidence in vaccines is contingent upon trust not only in the product itself but also in the providers, the health system, government and wider information environment that underpin vaccination decisions (3).  Another relevant reference(s) is needed, e.g.:  doi: 10.3390/ijerph20010712 | We have added an additional reference to support this point and strengthen the link between vaccine confidence and trust in health systems, providers, and the broader information environment. | 25 |
| Strengths and limitations of the study:  The core weakness of this study is its relatively small sample size; the same issue was also noted by other authors who conducted similar analyses. Other Ukrainian refugees, e.g., from other parts of Poland, may have different experiences with the Polish health system. | We have clarified in the limitations section that the study is based on a context-specific qualitative sample and is not intended to be statistically generalisable. We also note that experiences may differ across other populations and settings, including Ukrainian refugees in other parts of Poland. | 29 |
| A further weakness inherent to the study's format is its participant-snowball sampling system — the results obtained are therefore harder to generalize to the larger population. | We have added a clarification in the limitations section noting that snowball sampling was used to access participants in a conflict-affected setting, and that this may have influenced the composition of the sample and the perspectives captured. | 29 |
| The predominance of mothers is another limitation. Please discuss how it could influence the study results. | We have expanded the limitations section to reflect the predominance of mothers in the sample and note that this may shape the perspectives captured, with fathers and other caregivers underrepresented. | 29 |

We hope that these revisions have addressed the reviewers’ comments and improved the manuscript. We have also updated the Data Availability statement in line with PLOS Global Public Health requirements, including details on how data can be accessed in accordance with ethical and confidentiality constraints.

We would like to thank the editor and reviewers again for their valuable feedback and consideration of our work.

Kind regards,

Harriet Dwyer
