# Supplementary material for: “This is what a war does”- Trust, information ecosystems and childhood vaccination among Ukrainian parents: A qualitative study
Source: PLOS Glob Public Health. 2026 Jul 2;6(7):e0006742. doi: 10.1371/journal.pgph.0006742 (PMC13327253; doi:10.1371/journal.pgph.0006742)
Supplement: Peer review history

# Peer review: Decision Letter 0

**DOI:** 10.1371/journal.pgph.0006742.r001

**Authors:** Ryan Essex

5 Mar 2026

PGPH-D-25-03620

“This is what a war does”- The reshaping of trust, information and childhood vaccination among Ukrainian parents: a qualitative study

PLOS Global Public Health

Dear Dr. Dwyer,

Thank you for submitting your manuscript to PLOS Global Public Health. After careful consideration, we feel that it has merit but does not fully meet PLOS Global Public Health’s publication criteria as it currently stands. Therefore, we invite you to submit a revised version of the manuscript that addresses the points raised during the review process.

Please submit your revised manuscript by Apr 17 2026 11:59PM. If you will need more time than this to complete your revisions, please reply to this message or contact the journal office at globalpubhealth@plos.org. When you're ready to submit your revision, log on to https://www.editorialmanager.com/pgph/ and select the 'Submissions Needing Revision' folder to locate your manuscript file.

Please include the following items when submitting your revised manuscript:

- A letter that responds to each point raised by the editor and reviewer(s). You should upload this letter as a separate file labeled 'Response to Reviewers'.
- A marked-up copy of your manuscript that highlights changes made to the original version. You should upload this as a separate file labeled 'Revised Manuscript with Track Changes'.
- An unmarked version of your revised paper without tracked changes. You should upload this as a separate file labeled 'Manuscript'.

Guidelines for resubmitting your figure files are available below the reviewer comments at the end of this letter.

We look forward to receiving your revised manuscript.

Kind regards,

Ryan Essex

Academic Editor

PLOS Global Public Health

Journal Requirements:

1. Please include a complete copy of PLOS’ questionnaire on inclusivity in global research in your revised manuscript. Our policy for research in this area aims to improve transparency in the reporting of research performed outside of researchers’ own country or community. The policy applies to researchers who have travelled to a different country to conduct research, research with Indigenous populations or their lands, and research on cultural artefacts. The questionnaire can also be requested at the journal’s discretion for any other submissions, even if these conditions are not met.  Please find more information on the policy and a link to download a blank copy of the questionnaire here: https://journals.plos.org/globalpublichealth/s/best-practices-in-research-reporting. Please upload a completed version of your questionnaire as Supporting Information when you resubmit your manuscript.

1. Please send a completed 'Competing Interests' statement, including any COIs declared by your co-authors. If you have no competing interests to declare, please state "The authors have declared that no competing interests exist".

1. Please amend your detailed Financial Disclosure statement. This is published with the article. It must therefore be completed in full sentences and contain the exact wording you wish to be published.
  a. State the initials, alongside each funding source, of each author to receive each grant.
  b. State what role the funders took in the study. If the funders had no role in your study, please state: “The funders had no role in study design, data collection and analysis, decision to publish, or preparation of the manuscript.”

1. In the online submission form, you indicated that “Data made available upon reasonable request.”
  All PLOS journals now require all data underlying the findings described in their manuscript to be freely available to other researchers, either
  1. In a public repository,
  2. Within the manuscript itself, or
  3. Uploaded as supplementary information.
  This policy applies to all data except where public deposition would breach compliance with the protocol approved by your research ethics board. If your data cannot be made publicly available for ethical or legal reasons (e.g., public availability would compromise patient privacy), please explain your reasons by return email and your exemption request will be escalated to the editor for approval. Your exemption request will be handled independently and will not hold up the peer review process, but will need to be resolved should your manuscript be accepted for publication. One of the Editorial team will then be in touch if there are any issues.

If the reviewer comments include a recommendation to cite specific previously published works, please review and evaluate these publications to determine whether they are relevant and should be cited. There is no requirement to cite these works unless the editor has indicated otherwise.

Additional Editor Comments (if provided):

[Note: HTML markup is below. Please do not edit.]

Reviewers' comments:

Reviewer's Responses to Questions

**Comments to the Author**

1. Does this manuscript meet PLOS Global Public Health’s publication criteria? Is the manuscript technically sound, and do the data support the conclusions? The manuscript must describe methodologically and ethically rigorous research with conclusions that are appropriately drawn based on the data presented.

Reviewer #1: Yes

Reviewer #2: Yes

**********

2. Has the statistical analysis been performed appropriately and rigorously?

Reviewer #1: N/A

Reviewer #2: N/A

**********

3. Have the authors made all data underlying the findings in their manuscript fully available (please refer to the Data Availability Statement at the start of the manuscript PDF file)?

The PLOS Data policy requires authors to make all data underlying the findings described in their manuscript fully available without restriction, with rare exception. The data should be provided as part of the manuscript or its supporting information, or deposited to a public repository. For example, in addition to summary statistics, the data points behind means, medians and variance measures should be available. If there are restrictions on publicly sharing data—e.g. participant privacy or use of data from a third party—those must be specified.

Reviewer #1: Yes

Reviewer #2: Yes

**********

4. Is the manuscript presented in an intelligible fashion and written in standard English?

PLOS Global Public Health does not copyedit accepted manuscripts, so the language in submitted articles must be clear, correct, and unambiguous. Any typographical or grammatical errors should be corrected at revision, so please note any specific errors here.

Reviewer #1: Yes

Reviewer #2: Yes

**********

5. Review Comments to the Author

Please use the space provided to explain your answers to the questions above. You may also include additional comments for the author, including concerns about dual publication, research ethics, or publication ethics. (Please upload your review as an attachment if it exceeds 20,000 characters)

Reviewer #1: This manuscript demonstrates exceptional analytical depth in elucidating digital “trust chains,” particularly through its incisive depiction of Telegram’s transformation from a conduit for military alerts into a pivotal nexus for health information dissemination. This is powerfully substantiated by participant quotations, notably KII12’s emphasis on perceiving a “real person” behind Telegram channels, and reinforced by corroborative key-informant testimonies. The paper compellingly shows how perceived authenticity, immediacy and emotional connection now eclipse institutional authority in shaping trust during protracted crisis.

The methodological rigour is equally commendable. The collaborative framework integrating local Ukrainian and Polish researchers with UK-based scholars, together with the explicit use of a dynamic consent model, is a strong response to ethical complexity in conflict-affected settings and demonstrates sensitivity to power asymmetries and participant vulnerability. The reflexive field diary, clearly embedded in the analytic process, further enhances transparency and strengthens the credibility of the thematic analysis.

Theoretically, the study is well scaffolded through the use of Stuart Hall’s encoding–decoding framework and Henrik Vigh’s notion of “crisis as chronicity,” which together provide a robust interpretive lens for understanding how parents’ vaccination decisions become moral and agentic acts under existential precarity.

Targeted suggestions

Introduction

Consider foregrounding in the Introduction the persistence of approximately 15,000 zero-dose children in Ukraine (p. 7). While this statistic is presented later in the manuscript, introducing it earlier would sharpen the public-health urgency that frames the study.

Discussion

The paper would benefit from a more explicit interrogation of positionality, including outsider privilege and colonial epistemologies (p. 25). While reflexivity is addressed, making these dimensions more central would deepen methodological transparency and align with decolonial debates in global health research.

Conclusion

The findings on the pragmatic use of AI-mediated tools such as Google Translate and ChatGPT could be taken further by proposing their institutionalisation as adjunct public-health tools for displaced populations navigating bureaucratic and linguistic barriers (p. 12).

Re-emphasise the State Expert Centre’s transparent pharmacovigilance reporting as a cornerstone of rebuilding institutional trust, positioning it more clearly as a transferable policy model for other conflict-affected health systems (p. 19).

Overall, this is an empirically rich and theoretically sophisticated contribution that meaningfully advances understanding of trust, information ecosystems and vaccine confidence in war-affected contexts.

Reviewer #2: While I think this is a very important topic that warrants investigation, there were a few issues with the methodology and the manuscript itself that are significant enough that they undermine the contributions of the study. I have some reservations about this paper. They are outlined below.

Introduction

1.The Authors state:

Politically, low levels of trust in institutions, particularly towards governments and health systems, are significantly associated with reduced vaccine confidence.

and then cite the only one reference (5).

This is a very important observation that needs additional references to support. I would suggest adding references that concentrate specifically on Ukrainian migrants and refugees, such as:

doi: 10.1007/s00431-024-05847-2

doi: 10.2807/1560-7917.ES.2024.29.39.2400159.

doi: 10.1186/s12889-020-10105-9.

2.Please define the Research Gaps regarding the study’s main theme.

3.The manuscript would benefit from highlighting the study's novelty.

Methods

The authors state that “Ukraine has a long history of community hesitancy around vaccines” and cite only one relevant reference (23). Please consider citing more articles in the same vein; it is not mandatory, but here, you will find some interesting manuscripts that you can consider citing and adding to your references.

Vaccine Hesitancy in Ukraine: The Sign of a Crisis in Governance? | Wilson Center.

doi: 10.3390/healthcare13141744.

doi: 10.3389/fpubh.2024.1380627.

doi: 10.1186/s12889-020-10105-9.

doi: 10.3390/ijerph20010712.

They further state: As of July 2025, over 5.6 million refugees from Ukraine have been recorded globally, with nearly 5.1 million displaced Ukrainians recorded in Europe alone, three-quarters of whom are women and children (26). 3.3 million Ukrainians are internally displaced (26), and an estimated 4.8 million people remain in need of critical humanitarian assistance inside Ukraine and among Ukrainians in neighbouring countries (27).

Updating this information to the end of 2025 would be valuable.

Informed written consent was obtained from all participants. How did it work with participants interviewed online?

Interview topic guides were informed by the related literature and consultations with humanitarian responders currently working in Ukraine.

“the related literature” – references are important to add.

“consultations with humanitarian responders currently working in Ukraine”. How many of them helped to inform the interview topic guides?

My inclination is that the authors did not test the interview guide before launching it. What about the pilot study? Were any amendments made after piloting? If so, please describe. A brief description of all included topic guides and their attachment in the Appendix would be valuable.

What were the exclusion/inclusion criteria in this study?

Semi-structured interviews were conducted in person by the lead author and two research assistants who also acted as interpreters. What was their experience in conducting the semi-structured interviews with migrants? Which backgrounds did they represent? A variety of backgrounds allows for the reduction of potential desirability bias, regarding shaping of responses due to the role of the interviewer.

Were interviews with Ukrainian parents conducted in person or via Zoom? Where did all of the invited Ukrainian parents meet with the researchers for the interviews?

Key-informant interviews were conducted either in person or via Zoom. Who was the interviewer?

A reflexive field diary was maintained as a tool to navigate the research process, capturing contextual insights, methodological decisions, and the research team’s reflections on their positionality and interactions with participants. Was this a novel approach or had it been previously reported? Please specify.

Did Ukrainian participants represent adequate proportions for age, for participants with secondary/university degrees, and for having/not having a child to sufficiently explore the study objectives?

Why was the thematic analysis used in the study? What are the benefits of such analysis?

What actions helped researchers to proceed with an accurate qualitative analysis? Which steps were taken by the Authors until consensus could be achieved? E.g., intensive literature searches (textbook chapters and articles on qualitative research methods – which ones?), articles on qualitative studies regarding the main themes of the study – which ones?), discussions (conducted in which way?).

How were the data analyzed thematically?

Was the process of coding and development of themes inductive in nature, or did the authors plan beforehand to adhere to a pre-existing theoretical framework?

There is a repetition regarding the Ethics approval (pages 12 and 31). Please, modify.

Results

Figure 1: The authors analyzed “parents and caregivers”; however, according to Table 1, only parents were interviewed. Please clarify.

The quotation “Even if they [parents] can speak Polish, it’s preferable for them [ healthcare providers] to treat with Polish mothers.” is unclear. Please clarify.

“…several parents described having to pay out of pocket for certain vaccines, with costs reaching up to 700 zloty (approximately $193 USD) per dose…” Which parents? Ukrainian migrants in Warsaw or Ukrainians in Lviv? For which vaccines? Please clarify.

The Centre for Public Health, in partnership with humanitarian organisations like UNICEF and the World Health Organization with support from the World Bank, developed a system for preserving vaccines in conditions of frequent and unpredictable power outages. At the regional level, cold storage and transport systems were modernised, and primary health facilities received grants for backup generators for vaccine storage. Reference is needed to support this statement.

While regional hubs benefitted from upgraded infrastructure, remote and frontline communities continued to face challenges in vaccine delivery, health worker shortages and disrupted data systems. The movement of populations across Ukraine and outside the country further complicated vaccine management and monitoring.

Reference is needed to support this statement.

While also reflecting the interactions between mistrust and vaccination whereby confidence in vaccines is contingent upon trust not only in the product itself but also in the providers, the health system, government and wider information environment that underpin vaccination decisions (3).

Another relevant reference(s) is needed, e.g.:

doi: 10.3390/ijerph20010712

Strengths and limitations of the study:

The core weakness of this study is its relatively small sample size; the same issue was also noted by other authors who conducted similar analyses. Other Ukrainian refugees, e.g., from other parts of Poland, may have different experiences with the Polish health system.

A further weakness inherent to the study's format is its participant-snowball sampling system — the results obtained are therefore harder to generalize to the larger population.

The predominance of mothers is another limitation. Please discuss how it could influence the study results.

**********

6. PLOS authors have the option to publish the peer review history of their article (what does this mean?). If published, this will include your full peer review and any attached files.

**Do you want your identity to be public for this peer review?**  If you choose “no”, your identity will remain anonymous but your review may still be made public.

For information about this choice, including consent withdrawal, please see our Privacy Policy.

Reviewer #1: No

Reviewer #2: No

**********

[NOTE: If reviewer comments were submitted as an attachment file, they will be attached to this email and accessible via the submission site. Please log into your account, locate the manuscript record, and check for the action link "View Attachments". If this link does not appear, there are no attachment files.]

-->Figure Resubmissions:

While revising your submission, we strongly recommend that you use PLOS’s NAAS tool (https://ngplosjournals.pagemajik.ai/artanalysis) to test your figure files. NAAS can convert your figure files to the TIFF file type and meet basic requirements (such as print size, resolution), or provide you with a report on issues that do not meet our requirements and that NAAS cannot fix.

After uploading your figures to PLOS’s NAAS tool - https://ngplosjournals.pagemajik.ai/artanalysis, NAAS will process the files provided and display the results in the "Uploaded Files" section of the page as the processing is complete. If the uploaded figures meet our requirements (or NAAS is able to fix the files to meet our requirements), the figure will be marked as "fixed" above. If NAAS is unable to fix the files, a red "failed" label will appear above. When NAAS has confirmed that the figure files meet our requirements, please download the file via the download option, and include these NAAS processed figure files when submitting your revised manuscript.

# Author comment: Author response to Decision Letter 1

**DOI:** 10.1371/journal.pgph.0006742.r002

12 May 2026

Attachment Submitted filename: Response to reviewers.docx

# Peer review: Decision Letter 1

**DOI:** 10.1371/journal.pgph.0006742.r003

**Authors:** Ryan Essex, Ryan Essex

10 Jun 2026

“This is what a war does”- Trust, information ecosystems and childhood vaccination among Ukrainian parents: a qualitative study.

PGPH-D-25-03620R1

Dear Dwyer,

We are pleased to inform you that your manuscript '“This is what a war does”- Trust, information ecosystems and childhood vaccination among Ukrainian parents: a qualitative study.' has been provisionally accepted for publication in PLOS Global Public Health.

Before your manuscript can be formally accepted you will need to complete some formatting changes, which you will receive in a follow up email. A member of our team will be in touch with a set of requests.

Please note that your manuscript will not be scheduled for publication until you have made the required changes, so a swift response is appreciated.

IMPORTANT: The editorial review process is now complete. PLOS will only permit corrections to spelling, formatting or significant scientific errors from this point onwards. Requests for major changes, or any which affect the scientific understanding of your work, will cause delays to the publication date of your manuscript.

If your institution or institutions have a press office, please notify them about your upcoming paper to help maximize its impact. If they'll be preparing press materials, please inform our press team as soon as possible -- no later than 48 hours after receiving the formal acceptance. Your manuscript will remain under strict press embargo until 2 pm Eastern Time on the date of publication. For more information, please contact globalpubhealth@plos.org.

Thank you again for supporting Open Access publishing; we are looking forward to publishing your work in PLOS Global Public Health.

Best regards,

Ryan Essex

Academic Editor

PLOS Global Public Health

***********************************************************

Reviewer Comments (if any, and for reference):

Reviewer's Responses to Questions

**Comments to the Author**

1. If the authors have adequately addressed your comments raised in a previous round of review and you feel that this manuscript is now acceptable for publication, you may indicate that here to bypass the “Comments to the Author” section, enter your conflict of interest statement in the “Confidential to Editor” section, and submit your "Accept" recommendation.

Reviewer #2: All comments have been addressed

**********

2. Does this manuscript meet PLOS Global Public Health’s publication criteria? Is the manuscript technically sound, and do the data support the conclusions? The manuscript must describe methodologically and ethically rigorous research with conclusions that are appropriately drawn based on the data presented.

Reviewer #2: Yes

**********

3. Has the statistical analysis been performed appropriately and rigorously?

Reviewer #2: I don't know

**********

4. Have the authors made all data underlying the findings in their manuscript fully available (please refer to the Data Availability Statement at the start of the manuscript PDF file)?

The PLOS Data policy requires authors to make all data underlying the findings described in their manuscript fully available without restriction, with rare exception. The data should be provided as part of the manuscript or its supporting information, or deposited to a public repository. For example, in addition to summary statistics, the data points behind means, medians and variance measures should be available. If there are restrictions on publicly sharing data—e.g. participant privacy or use of data from a third party—those must be specified.

Reviewer #2: Yes

**********

5. Is the manuscript presented in an intelligible fashion and written in standard English?

PLOS Global Public Health does not copyedit accepted manuscripts, so the language in submitted articles must be clear, correct, and unambiguous. Any typographical or grammatical errors should be corrected at revision, so please note any specific errors here.

Reviewer #2: Yes

**********

6. Review Comments to the Author

Please use the space provided to explain your answers to the questions above. You may also include additional comments for the author, including concerns about dual publication, research ethics, or publication ethics. (Please upload your review as an attachment if it exceeds 20,000 characters)

Reviewer #2: Dear Authors,

Congratulations for your good work.

**********

7. PLOS authors have the option to publish the peer review history of their article (what does this mean?). If published, this will include your full peer review and any attached files.

**Do you want your identity to be public for this peer review?**  If you choose “no”, your identity will remain anonymous but your review may still be made public.

For information about this choice, including consent withdrawal, please see our Privacy Policy.

Reviewer #2: **Yes:** Maria Ganczak

**********
